# Human immune cells infiltrate the spinal cord and impair recovery after spinal cord injury in humanized mice

**DOI:** 10.1038/s41598-019-55729-z

**Published:** 2019-12-13

**Authors:** Randall S. Carpenter, Roselyn R. Jiang, Faith H. Brennan, Jodie C. E. Hall, Manoj K. Gottipati, Stefan Niewiesk, Phillip G. Popovich

**Affiliations:** 1 Neuroscience Graduate Program, The Ohio State University, Columbus, Ohio USA; 2 Belford Center for Spinal Cord Injury, The Ohio State University, Columbus, Ohio USA; 3 Center for Brain and Spinal Cord Repair, The Ohio State University, Columbus, Ohio USA; 4 Department of Neuroscience, The Ohio State University, Columbus, Ohio USA; 5 Department of Veterinary Biosciences, The Ohio State University, Columbus, Ohio USA

**Keywords:** Spinal cord injury, Neuroimmunology, Neuroimmunology

## Abstract

Humanized mice can be used to better understand how the human immune system responds to central nervous system (CNS) injury and inflammation. The optimal parameters for using humanized mice in preclinical CNS injury models need to be established for appropriate use and interpretation. Here, we show that the developmental age of the human immune system significantly affects anatomical and functional outcome measures in a preclinical model of traumatic spinal cord injury (SCI). Specifically, it takes approximately 3–4 months for a stable and functionally competent human immune system to develop in neonatal immune compromised mice after they are engrafted with human umbilical cord blood stem cells. Humanized mice receiving a SCI before or after stable engraftment exhibit significantly different neuroinflammatory profiles. Importantly, the development of a mature human immune system was associated with worse lesion pathology and neurological recovery after SCI. In these mice, human T cells infiltrate the spinal cord lesion and directly contact human macrophages. Together, data in this report establish an optimal experimental framework for using humanized mice to help translate promising preclinical therapies for CNS injury.

## Introduction

Immunocompromised mice engrafted with human immune systems (i.e. “humanized” mice) are powerful pre-clinical models for studying human immune cell function. These mice provide translational value by revealing underlying pathophysiology after injury and disease, and by allowing the *in vivo* testing of novel treatment strategies. Previously, we documented the feasibility of using humanized mice to study systemic and neuroinflammatory changes caused by traumatic spinal cord injury (SCI)^1^. That report, while the first of its kind, was a feasibility study that did not provide a comprehensive analysis of the composition or function of human immune cells or how these parameters change as a function of time post-engraftment.

Developmental effects on human immune composition and responsiveness to stimuli are not clearly discussed in the humanized mouse literature and existing data are conflicting. For instance, some data indicate that in humanized mice, both innate and adaptive human immune cells exhibit functional responses to inflammatory stimuli (e.g., proliferation, cytokine production, antibody synthesis, migration toward chemotactic cues, etc.)^2–12^. However, other data indicate that human immune cells develop in humanized mice but their functions are impaired^13–16^. Questions about the functional competency of human immune cells in this model prompted the development of “next-generation” humanized mouse models with improved immune function are being generated to address supposed issues^17–23^. These conflicting data could be explained, in part, by variability in the maturation state of human immune cells. Indeed, recent reports show that human immune cell functions in humanized mice vary as a function of time post-engraftment^6,24–26^. A delay of human immune cell development in humanized mice is logical if one considers that in normal mice, immune system development begins *in utero*^27^. The humanized mouse model requires that an immune compromised host receive injections of undifferentiated human umbilical cord blood (UCB) stem cells. The integration and growth of these stem cells in the mouse host takes time; they must engraft the bone marrow and secondary lymphoid tissues (e.g., spleen, lymph nodes) then differentiate into multi-lineage human progenitors before organizing into functional immune niches that generate mature human immune cells.

In rodent SCI models, most experiments are performed in young adult animals at ~8–12 weeks of age. We predicted that if humanized mice are injured at that age, as we have done previously^1^, their human immune systems will be immature and functionally distinct from humanized mice in which longer periods of development were allowed after engraftment with human UCB stem cells. To test this hypothesis, we compared the composition and relative frequency of human peripheral blood leukocytes (PBLs) as a function of time post-engraftment in NOD-SCID-IL2rγ^null^ (NSG) mice. New data in this report show that it takes 3–4 months for human UCB stem cells to generate a robust human immune system in NSG mice. By 4 months, both human innate and adaptive immune cells form proliferative niches and in response to inflammatory stimuli, they produce antibodies and release cytokines indicating that they are functional. A comparative analysis of two humanized mouse cohorts at different post-engraftment intervals – one at 2 months the other at 4 months – reveals significant differences in how the human immune system responds to an experimental model of traumatic SCI. When SCI is performed in mice with a fully developed human immune system, lesion pathology is worse and hindlimb functional recovery is impaired relative to non-engrafted and age matched NSG mice. Together, these novel data reveal an optimal experimental framework for using humanized mice to study human immune responses to acute CNS trauma. These data also illustrate the adverse effects that post-injury activation of the human immune system can have on lesion pathology and recovery of neurologic function after SCI.

## Results

### Human immune cell engraftment and composition is optimal by 4 months post-engraftment

**Figure 1:**
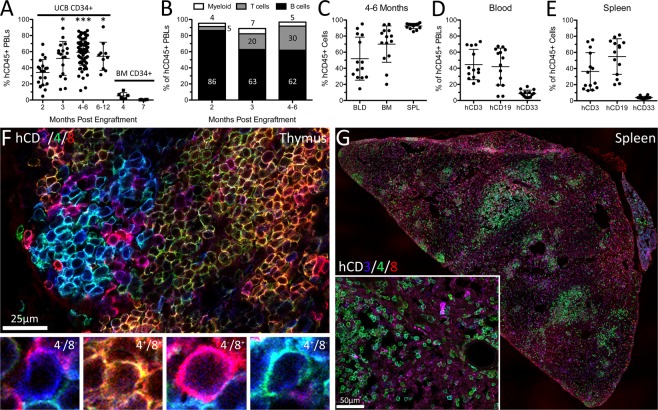
Long-lived engraftment of human immune systems in NSG mice occurs without toxicity. Human immune systems were derived from hCD34^+^ umbilical cord blood stem cells. (**A**) Engraftment efficiency in mice is affected by time post-engraftment and source of stem cells. UCB = umbilical cord blood, BM = bone marrow. Data average ± SD; *p < 0.05 ***p < 0.001 compared to 2 months post-engraftment (UCB CD34^+^ only), one-way ANOVA with Tukey’s multiple comparison test. (**B**) Proportion of human CD45^+^ peripheral blood leukocyte (PBL) subsets. (**C**) Human CD45^+^ cells in the blood (BLD), bone marrow (BM), and spleen (SPL) of hNSG mice 4–6 months post-engraftment. (**D**,**E**) Human immune subsets (hCD3, hCD19, and hCD33) in blood (**D**) and spleen (**E**) of hNSG mice 4–6 months post-engraftment. (**F**,**G**) Human CD3^+^, CD4^+^, and CD8^+^ T cells identified in thymus (**F**) and spleen (**G**) of naïve hNSG mice 4 months post-engraftment. Classical human CD3^+^ T cell subsets in thymus are highlighted (**F**), including double negative CD4/CD8 (blue), double positive CD4/CD8 (yellow-orange), and single positive CD4 (green) and CD8 (pink).

To determine when peak development of a human immune system occurs in humanized mice, we quantified the percentage of human peripheral blood lymphocytes (hPBLs) up to 12 months after intrahepatic injection of human UCB CD34^+^ stem cells into NOD-SCID-IL2rγ^null^ (NSG) mouse pups (see Methods)^1,28^. At two months post-engraftment, human CD45^+^ (hCD45^+^) PBLs comprised 34% of total circulating leukocytes (Fig. 1A), increasing to 59% at 4 months post-engraftment. From 4–12 months post-engraftment, hPBLs were stable. In most hNSG mice, 30–90% human chimerism was achieved. Human chimerism was poor in only 5% (n = 4/78) of hNSG mice, as defined by <10% hCD45^+^ PBLs at 4 months post-engraftment.

In parallel with a plateau in the frequency of hPBLs, we noted a time-dependent change in the composition of circulating human immune cells (Fig. 1B,D). At 2 months post-engraftment, most human immune cells were CD19^+^ B cells (86%). At 4–6 months post-engraftment, the frequency of hCD19^+^ B cells decreased to 62%, concomitant with an increase in human CD3^+^ (hCD3^+^) T cells (30% of hPBLs). Human myeloid cells (hCD33^+^) comprised the smallest proportion of human cells from 2–6 months post-engraftment (4–7%).

By 4 months, when the frequency of circulating human PBLs reached a plateau, robust human immune cell engraftment was also observed in the bone marrow and spleen (Fig. 1C,E; Supplemental Fig. 3A,B). Human immune composition in the spleen matches the blood (Fig. 1D,E), with many human lymphocytes (T and B lymphocytes) and few myeloid cells. In the thymus of hNSG mice, mixed populations of hCD4^−^/hCD8^−^, hCD4^+^/hCD8^+^, hCD4^−^/hCD8^+^, and hCD4^+^/hCD8^−^ T cells could be found indicating ongoing thymopoiesis (Fig. 1F). NSG mice not injected with human stem cells had negligible thymus development. Spleens of humanized mice contained mostly human immune cells (Fig. 1C) with the white pulp densely populated by hCD3^+^/hCD4^+^ human T cells (Fig. 1G).

The source of human stem cells was also important. Compared to the robust chimerism that was achieved using UCB stem cells, adult human bone marrow CD34^+^ stem cells were inefficient as a donor source. At 4 months post-engraftment with adult bone marrow stem cells, >10% hCD45^+^ PBLs was achieved in only 1/6 mice and human chimerism was <1% in all mice by 7 months post-engraftment (Fig. 1A).

Collectively, these data illustrate that hNSG mice do not develop a mature human immune system until 3–4 months post-engraftment and that human UCB CD34^+^ stem cells are an optimal donor source. These are important biological variables that are not well-documented in the literature but if ignored, could dramatically affect experimental outcome^3,6,8,24,25^.

### Human immune cells respond to *ex vivo* and *in vivo* immune stimulation

**Figure 2:**
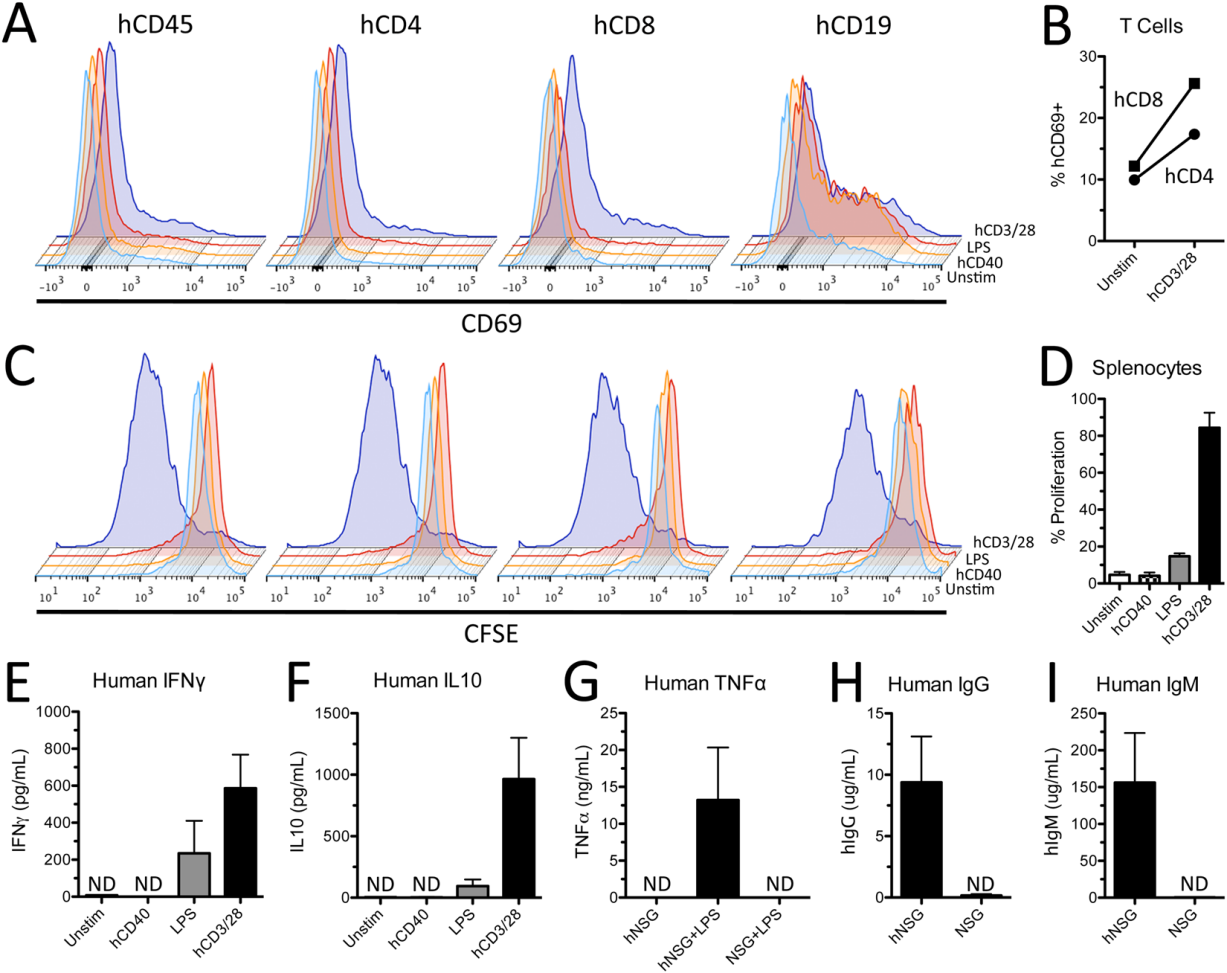
Human innate and adaptive immune cells from hNSG mice are functional and respond to cell-specific stimulation. (**A**) Human splenocytes upregulate cell surface expression of activation marker CD69 48 hours after stimulation with human CD3/28 antibody and rhIL2. (**B**) Proportion of hCD4^+^ and hCD8^+^ T cells expressing CD69 48 hours after stimulation by hCD3/28 and rhIL2. (**C**) Decrease in CFSE staining demonstrating robust proliferation of human splenocytes stimulated with hCD3/28 and rhIL2. (**D**) Proportion of proliferating splenocytes 96 hours after cell specific stimulation. (**E**,**F**) Quantification of human interferon gamma (IFNγ) and IL10 in culture supernatants after 96 hours of cell specific stimulation. (**G**) Human TNFα quantification in blood serum 1 hour after *in vivo* injection with 3 mg/kg lipopolysaccharide (LPS). Human IgG (**H**) and IgM (**I**) from blood serum in hNSG mice. Note the absence of human cytokines and antibodies in blood serum of non-engrafted NSG mice treated with LPS, demonstrating species specificity of ELISAs. ND = not detected. Data average ± SEM; n = 2 biological replicates in (**B**,**D**) n = 4 biological replicates in (**E**,**F**) n = 3 mice per group in (**G**,**H**) n = 3 NSG and n = 6 hNSG mice in (**I**,**J**).

To determine whether human immune cells in hNSG mice are functional by 4 months post-engraftment, human splenocytes were isolated, purified (see Supplemental Fig. 4A) and then activated *ex vivo* using cell-specific stimuli. Human splenocytes were comprised mostly of hCD4^+^ T cells, hCD19^+^ B cells and hCD8^+^ T cells (Supplemental Fig. 4B). In response to polyclonal stimulation with hCD3/28 and recombinant human IL2 (rhIL2), human T cells increased expression of hCD69 (Fig. 2A,B), a cell activation marker, accompanied by robust proliferation (Fig. 2C,D; Supplemental Fig. 4C) and production of human IFNγ and IL-10 (Fig. 2E,F).

When the same cell suspensions were exposed to hCD40 activating antibody (clone 5C3) and rhIL4, i.e., B cell-specific stimuli, human B cells increased their expression of hCD69 (Fig. 2A,B) but they did not proliferate or produce cytokines (Fig. 2C–F). Just as in normal humans or mice, full activation of B cells required T cell help; when purified human splenocyte suspension were stimulated with with hCD3/28 and rhIL2, robust hCD19^+^ B cell proliferation was induced (Fig. 2C).

Lipopolysaccharide (LPS), a canonical activator of toll-like receptor 4 (TLR4) found mostly on myeloid cells, also increased proliferation and production of human cytokines by human splenocytes (Fig. 2C–F). Similarly, LPS injected *in vivo* (3 mg/kg, i.p.) elicited production of human TNFα (hTNFα) by 1-hour post-injection (Fig. 2G). Human TNFα was not detected in serum from naive hNSG mice or non-humanized NSG mice injected with LPS. We also detected human IgG and IgM antibodies in blood from hNSG mice but not non-humanized NSG mice (Fig. 2H,I). Together, these data prove that human immune cells from hNSG mice are functional; they respond *ex vivo* and *in vivo* to physiologically-relevant stimuli, producing a range of immune effector molecules (e.g., cytokines, antibodies).

### Time post-engraftment determines human peripheral immune cell responses to contusive SCI

**Figure 3:**
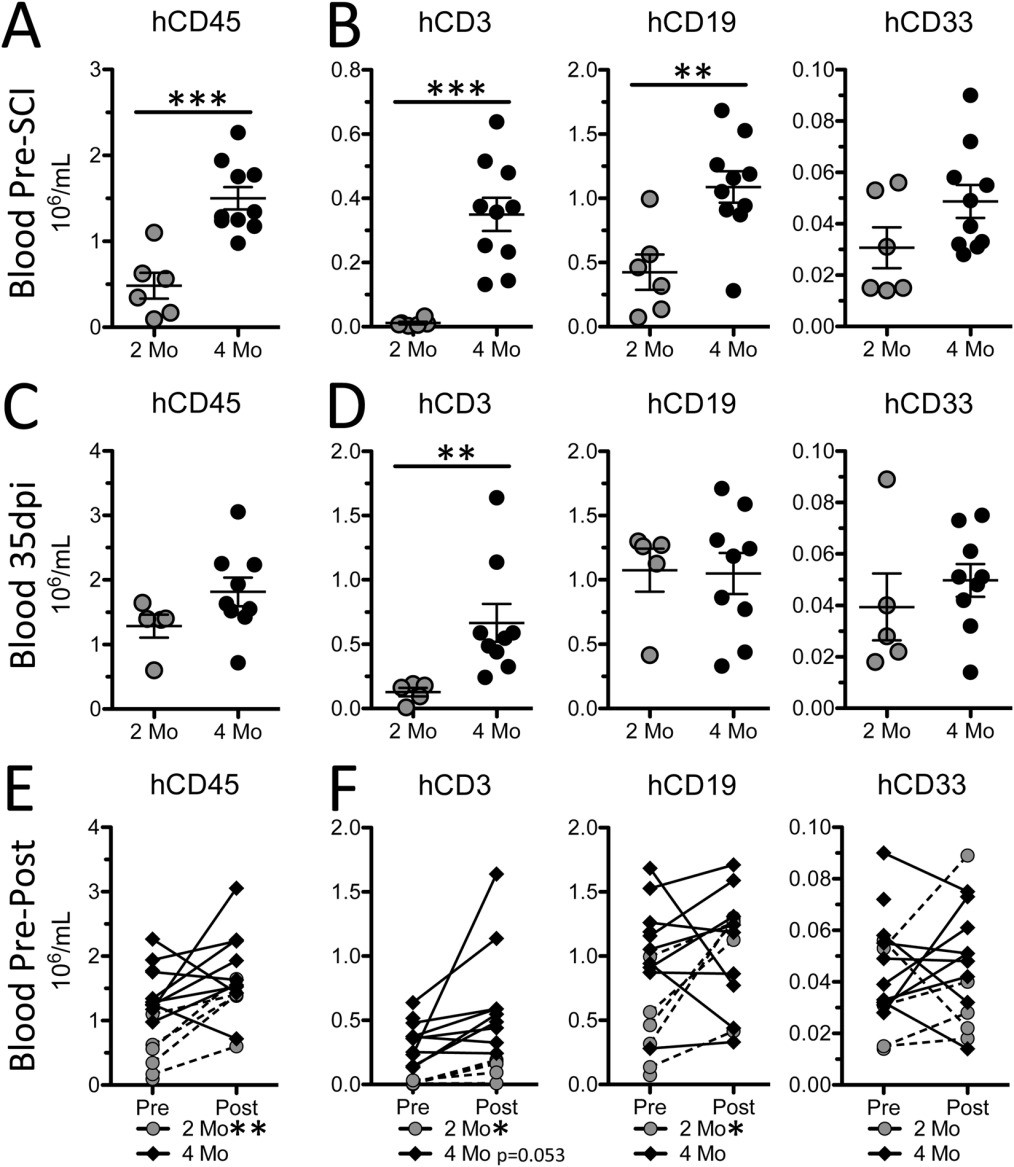
Human peripheral blood leukocyte responses to SCI differ as a function of time post-engraftment. Total numbers of human PBLs (**A**,**C**), and human PBL subsets (**B**,**D**), 7 days prior to and 35 days after SCI. Change in numbers of human PBLs (**E**), and human PBL subsets, from pre- to post-SCI. *p < 0.05 **p < 0.01 ***p < 0.001 student’s unpaired (**A**–**D**) and paired (**E**,**F**) t-test. Data average ± SEM.

Data in Figs. 1–2 indicate that the composition, relative density and functional maturity of human immune cells increases as a function of time post-engraftment in humanized mice. Thus, one would predict that the effects of SCI on immune system activation and the subsequent effects of neuroinflammation and lesion histopathology will change as a function of time post-engraftment. To test this hypothesis, a single human UCB donor was used to generate two age-matched cohorts of hNSG mice. Both groups received a SCI at the same time – one at 2 months post-engraftment and the other at 4 months post-engraftment. Both cohorts survived to 35 days post-injury (dpi). Consistent with data from Fig. 1, uninjured hNSG mice had more human PBLs at 4 months post-engraftment than at 2 months (Supplemental Fig. 5A) – all circulating human leukocyte subsets including T lymphocytes (hCD3), B lymphocytes (hCD19) and myeloid cells (hCD33) increased in number and proportion as a function of time post-engraftment (Fig. 3B**;** Supplemental Fig. 5B).

**Figure 4:**
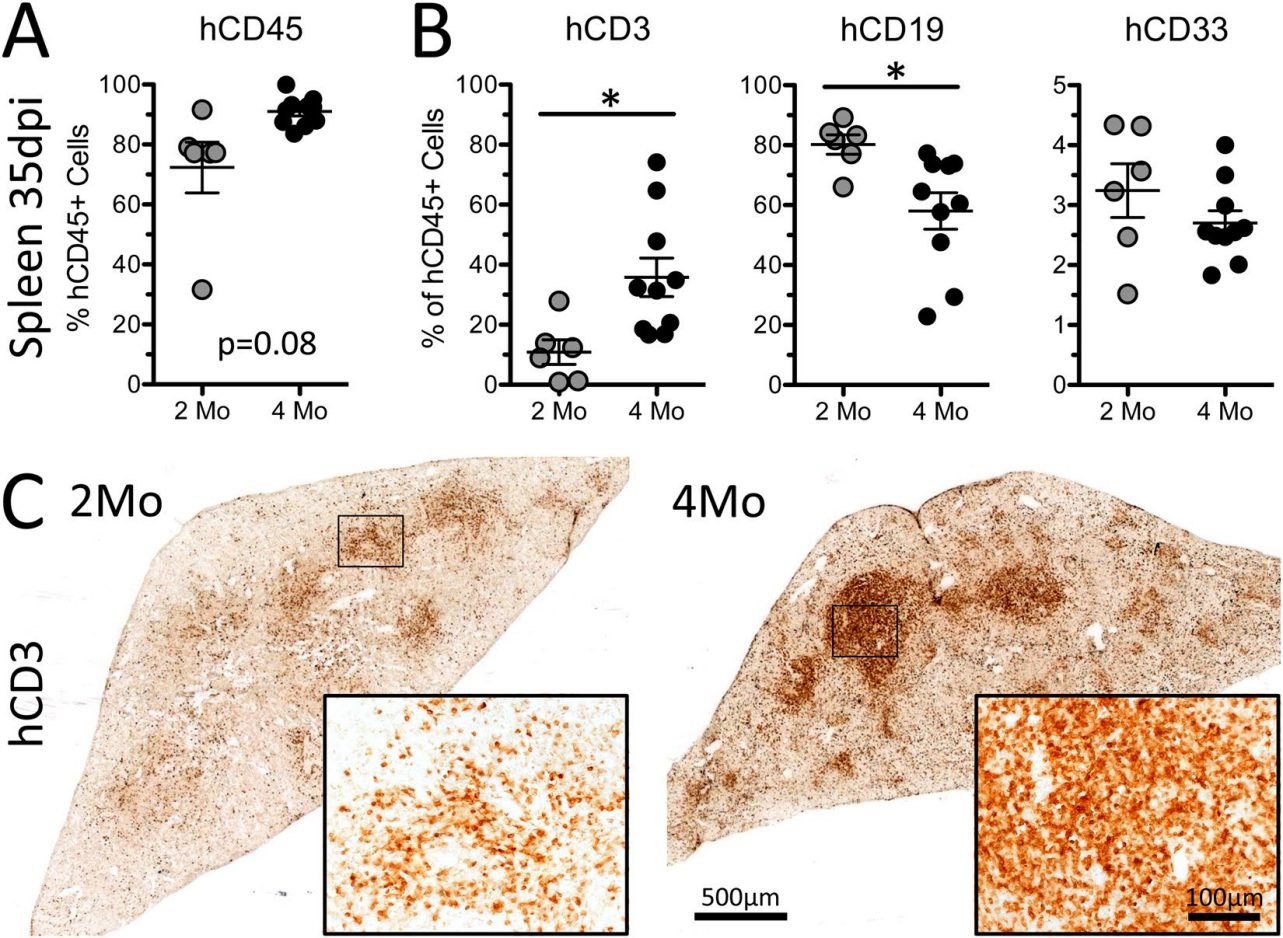
Human splenocyte proportion and composition 35 days after SCI in hNSG mice differ as a function of time post-engraftment. Human splenocyte chimerism (**A**) and composition (**B**) at 35 dpi between hNSG mice injured at 2- and 4-months post-engraftment. (**C**) Human CD3^+^ T cells in spleens of hNSG cohorts. Images are representative of the mean hCD3 values for both groups. *p < 0.05 student’s t-test. Data average ± SEM.

Five weeks after SCI we noted marked differences in circulating leukocyte responses between the two cohorts. In the 2-month cohort, total numbers of circulating human leukocytes increased after SCI (Fig. 3E,F**;** Supplemental Fig. 5E,F. This was likely an effect of continued maturation of the human immune system rather than a direct result of SCI. Indeed, by 35 dpi cells from mice in the 2-month cohort had been engrafted >3 months with the relative proportion of hCD45^+^ PBLs in most mice being about the same as in uninjured humanized mice at 3 months post-engraftment (**compare** Fig. 1A,B) and similar to pre-injury values found in the 4-month post-engraftment cohort (**compare** Fig. 3A,C**;** Supplemental Fig. 5A,C). Also, post-injury changes in hCD45^+^ PBLs were modest in the 4-month post-engraftment cohort (Fig. 4E) and were marked by a selective increase in the proportion of T cells (Fig. 3F**;** Supplemental Fig. 5F).

Given the importance of the spleen in regulating neuro-immune interactions after SCI^29,30^, we quantified human splenocyte subsets in both cohorts 35 dpi (Fig. 4A,B). The relative proportions of human T, B, and myeloid cells in spleen mirrored their proportions in the blood 35 dpi, which is consistent with the role of the spleen as a reservoir for circulating human leukocytes. Spleens of both groups displayed preferential clustering of hCD3^+^ T cells in the splenic white pulp. Again, the relative density of T cell clusters was increased in the 4-month post-engraftment cohort (Fig. 4C).

### Time post-engraftment determines the magnitude and composition of human T cell infiltration into the injured spinal cord

**Figure 5:**
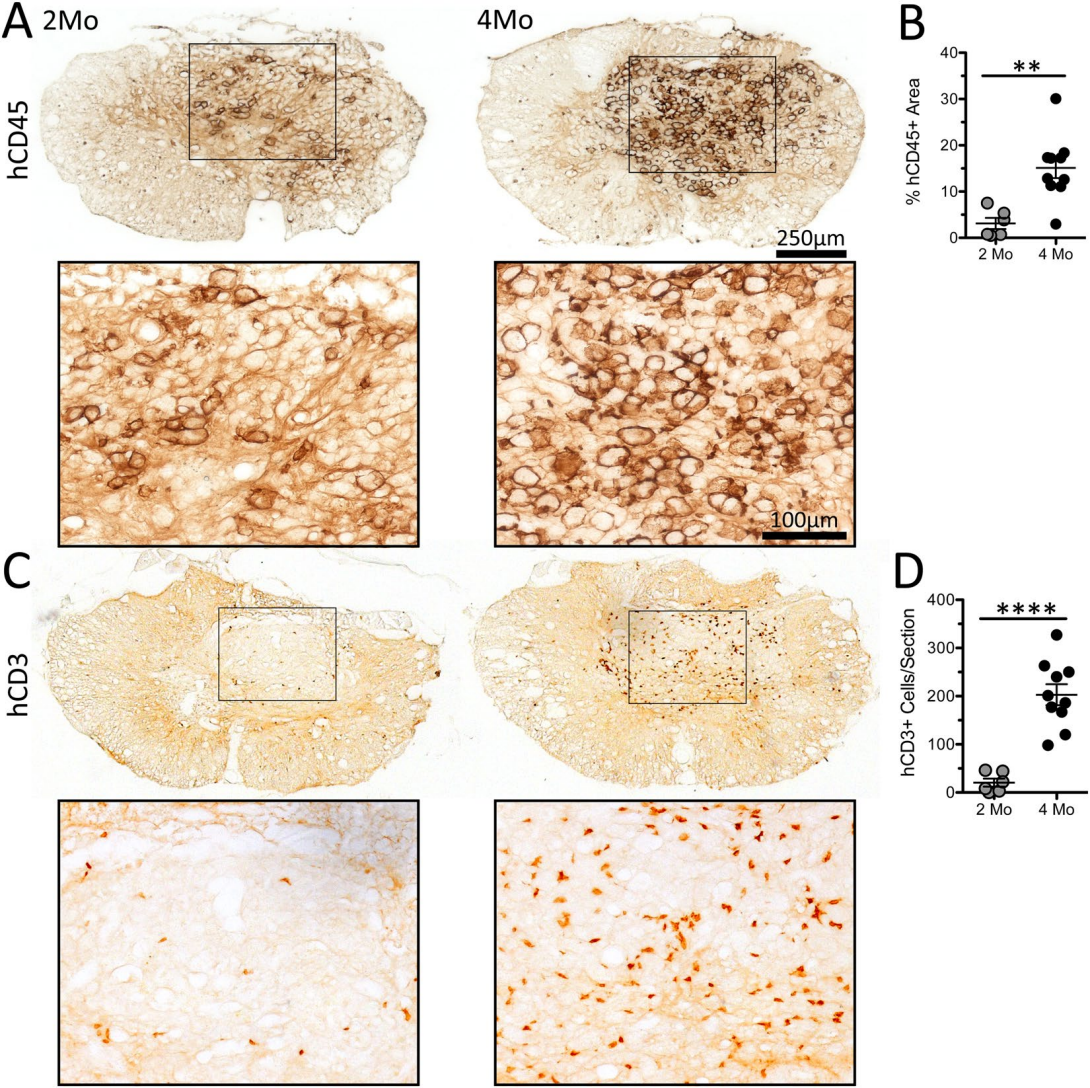
Time post-engraftment determines the magnitude of human immune cell infiltration into the injured spinal cord of hNSG mice. (**A**) Human CD45^+^ leukocytes within the lesion epicenter, primarily confined to the central lesion. (**B**) Proportional area analysis of hCD45^+^ immunolabeling within the lesion. (**C**) Human CD3^+^ T cells within the lesion epicenter, with quantification (**D**). Images representative of the mean for each data set. **p < 0.01 ****p < 0.0001 student’s t-test. Data average ± SEM.

Previously, we showed that SCI in humanized mice elicits neuroinflammation in the injured spinal cord, comprised mostly of mouse microglia, human macrophages and human lymphocytes^1^. To determine whether time post-engraftment affects the magnitude of human neuroinflammation and the ability of human immune cells to influence spinal cord anatomy, we compared recruitment of human leukocytes in the 2- and 4-month cohorts. Infiltration by hCD45^+^ leukocytes was increased in SCI mice from the 4-month cohort (Fig. 5A,B). Additionally, hCD3^+^ T cells were increased ~10-fold in mice injured at 4 months post-engraftment compared to 2 months post-engraftment (Fig. 5C,D).

**Figure 6:**
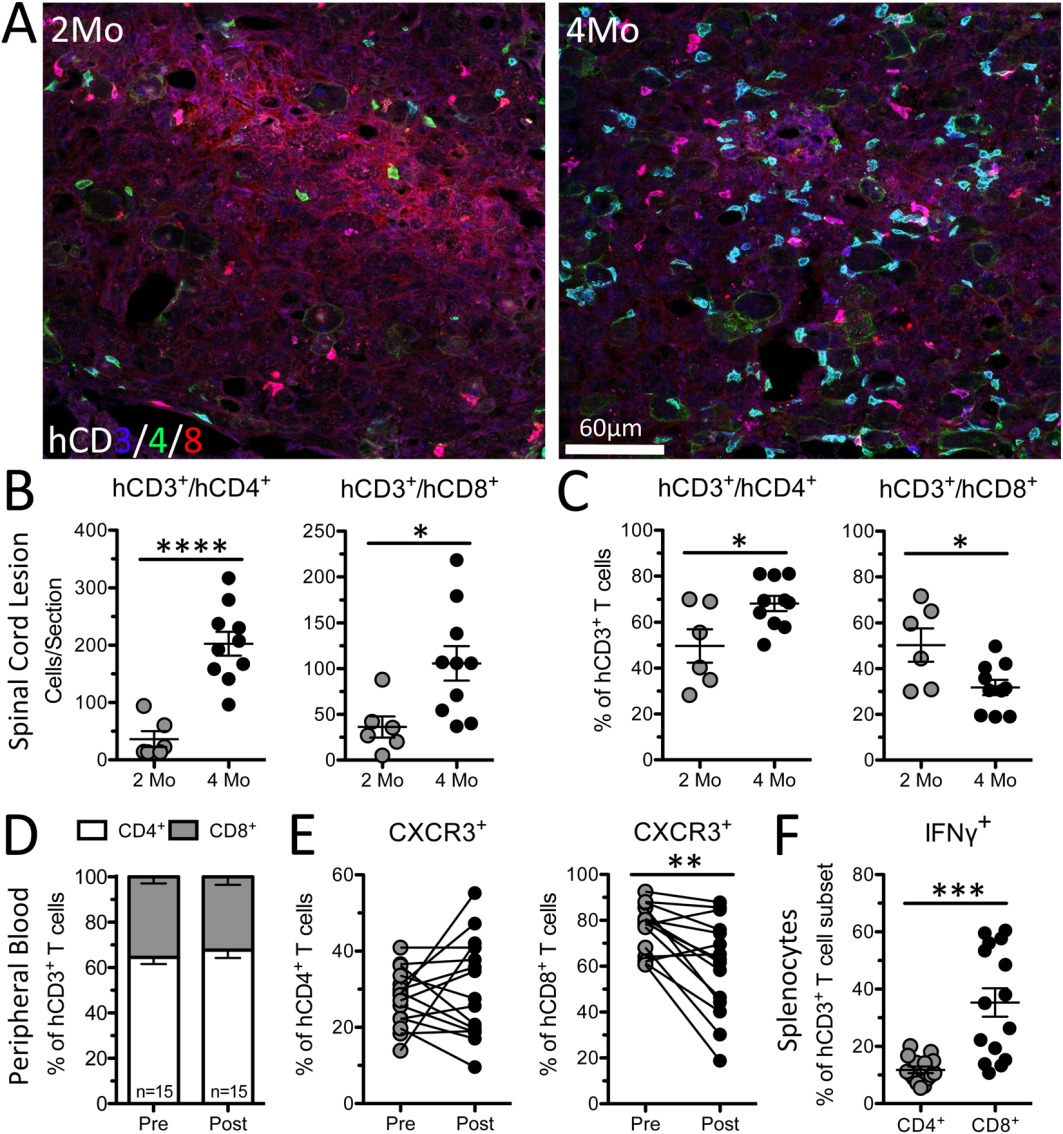
Intraspinal human helper and cytotoxic T cell subsets increase with time post-engraftment, reflect proportions of human T cell subsets in blood, and are functional after SCI. (**A**) Immunofluorescent labeling of hCD3^+^/hCD4^+^ helper and hCD3^+^/hCD8^+^ cytotoxic T cells at the lesion epicenter 35 dpi in hNSG mice injured at 2- and 4-month post-engraftment. MIPAR automated quantification of total numbers (**B**) and relative proportion (**C**) of human T cell subsets. (**D**) Proportion of hCD4^+^ and hCD8^+^ T cells in peripheral blood before SCI and 35 dpi. (**E**) Proportion of CXCR3^+^ human T cells (hCD4^+^ and hCD8^+^) in peripheral blood before SCI and 35 dpi. (**F**) Proportion of splenic IFNγ^+^ human T cells after stimulation with PMA/Ionomycin from hNSG mice 35 dpi. *p < 0.05 **p < 0. 01 ***p < 0.001 ****p < 0.0001 student’s t-test (**B**,**C**,**F**) and paired student’s t-test (**D**,**E**). Data average ± SEM.

Multi-color immunofluorescent confocal microscopy identified hCD3^+^/hCD4^+^ helper and hCD3^+^/hCD8^+^ cytotoxic T cell subsets within spinal cord lesions (Fig. 6A). We never observed colocalization of hCD4^+^ and hCD8^+^ labeling on any single hCD3^+^ T cell. Consistent with blood and spleen data in Figs. 3–4, more human T cell subsets were found in the injured spinal cord of hNSG mice from the 4-month cohort (Fig. 6B). The ratio of helper to cytotoxic T cells in spinal cord lesions of hNSG mice injured at 4 months post- engraftment (70%:30%) was significantly greater than at 2 months (50%:50%) (Fig. 6C) and matched the proportion of CD4/CD8 T cells found in peripheral blood (Fig. 6D). Changes in the ratio of CD4:CD8 T cells could indicate differences in T cell activation and their ability to gain entry into sites of inflammation. Indeed, the relative expression of the chemokine receptor CXCR3, which is highly expressed on effector T cells and plays an important role in T cell trafficking and function, decreased in CD8^+^ T cells after SCI (Fig. 6E). When human T cells were isolated from the spleen of hNSG mice at 35 dpi, both subsets produced IFNγ in response to stimulation, with more cytotoxic human T cells expressing IFNγ than helper T cells (Fig. 6F).

**Figure 7:**
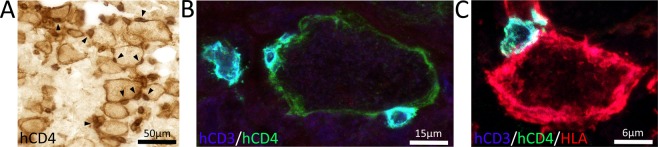
Human T helper subsets directly contact human macrophages. (**A**) Immunolabeling of hCD4 identifies both small and large cells with morphology of T cells and macrophages, respectively. Small hCD4^+^ T cells were often found directly adjacent to large, phagocytic human macrophages, indicating T cell-macrophage interaction within the injured spinal cord (arrowheads). (**B**) Confocal imaging of hCD3^+^/CD4^+^ T cells directly contacting a hCD3^-^/hCD4^+^ human macrophage. (**C**) Confocal imaging of a hCD3^+^/CD4^+^ T cell directly contacting a human HLA^+^ human macrophage.

Our previous study showed that large, phagocytic human macrophages populate the injured spinal cord in humanized mice^1^. Here, we expanded our phenotypic analyses of these cells. In the injured spinal cord of both hNSG cohorts, a subset of large (presumably activated phagocytic) human macrophages expressed hCD4 on their cell membrane (Fig. 7A,B). Although CD4 selectively labels helper T lymphocytes in mice, it is expressed by human and rat macrophages^31–39^. In the injury epicenter, human helper T cells (hCD3^+^/hCD4^+^) were often found in direct contact with hCD4^+^ macrophages (Fig. 7A,B). These T cell-macrophage interactions occurred in both hNSG cohorts but appeared to be more frequent in the 4-month cohort. Importantly, these human macrophages expressed human leukocyte antigen complexes (HLA-DR/DQ/DP), necessary for engaging with and activating antigen-dependent T lymphocytes (Fig. 7C). Together, data in Figs. 5–7 indicate that the composition of human leukocytes recruited to the injured spinal cord includes functional human T cells and macrophages and that recruitment matures as a function of time post-engraftment. Ultimately, the composition of the human neuroinflammatory lesions in the humanized mouse model is indistinguishable from that described after SCI in conventional mice, rats and humans^32,40–46^.

### A human immune system in NSG mice worsens lesion pathology and hindlimb functional recovery after SCI

Post-injury recruitment of T lymphocytes is a regular component of the neuroinflammatory reaction in mouse, rat and human^32,40–46^. In pre-clinical mouse and rat models of CNS injury, both injurious and protective effects have been attributed to infiltrating T cells after SCI^41–43,47–57^. For obvious reasons, establishing a causal role for infiltrating T cells in either injury or repair has not been possible in people with SCI.

**Figure 8:**
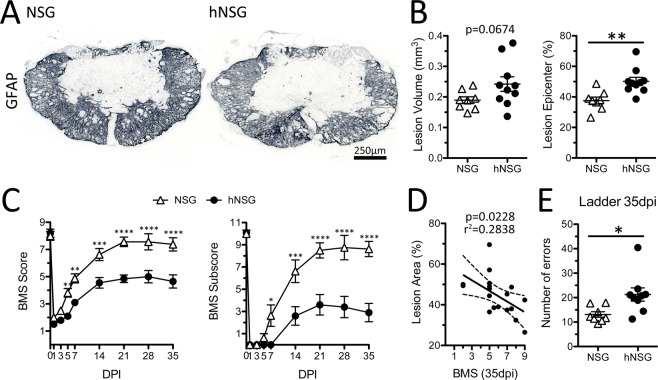
Humanized NSG mice (4-months post-engraftment) have increased lesion pathology and impaired functional recovery after SCI compared to age-matched non-engrafted NSG mice. (**A**) Astrocyte (GFAP) labeling of injury epicenter. (**B**) Lesion volume and area (proportion) at epicenter as defined as GFAP-negative region. (**C**) BMS score and subscore from 1–35 dpi. (**D**) Linear regression analysis of lesion size and BMS scores at 35 dpi. (**E**) Number of errors (foot falls) on a horizontal ladder test 35 dpi. *p < 0.05 **p < 0.01 ***p < 0.001 ****p < 0.0001 student’s t-test (**B**,**F**) and repeated measures two-way ANOVA with Bonferroni multiple comparisons test (**C**). Data average ± SEM.

Previously, we found that lesion pathology was exacerbated and functional recovery was impaired in hNSG mice as compared to non-engrafted immunocompromised NSG control mice^1^. However, our previous study occurred before we understood the importance of post-engraftment timing on maturation of the human immune system in hNSG mice and did not incorporate age-matched non-engrafted NSG mice as controls. Here, we compared lesion pathology and functional recovery in non-engrafted NSG mice and age-matched hNSG mice with mature (4-months post-engraftment) human immune systems after spinal contusion injury. hNSG mice develop larger lesions (Fig. 8A,B) with significantly worse recovery of hindlimb function as defined by open field locomotor analysis (Fig. 8C) and foot falls on the horizontal ladder test (Fig. 8E). Worse hindlimb function at 35 dpi correlated with an increase in lesion size (Fig. 8D). These data indicate that post-injury activation of human immune cells, like mouse immune cells, can exacerbate lesion pathology and impair neurological recovery after SCI^1^.

## Discussion

The anatomical, functional and hematological consequences of a contusive spinal cord injury (SCI) in humanized NSG (hNSG) mice were previously described by us^1^. Data in that report were the first to illustrate the feasibility of using humanized mice to test hypotheses related to neuro-immune interactions after SCI. However, those feasibility data did not take into consideration the importance of post-engraftment time on human immune cell composition and function.

Data in this report show that the relative efficiency of human chimerism and corresponding changes in human immune cell composition change as a function of time post-engraftment. Human chimerism peaks between 3–4 months post-engraftment and remains stable for at least 12 months. Although we did not measure white blood cell subsets in “normal” (e.g., C57 BL6/J) mice, data available in peer-reviewed publications^58^ and also from commercial vendors indicate 80–90% of peripheral blood leukocytes in C57BL/6J mice are T and B lymphocytes, similar to hNSG mice. Of the human immune cells that we characterized, T lymphocytes require longer developmental times and this development is pivotal for complete maturation of the human immune system in hNSG mice. In humanized mice, human T cells help promote the maturation and functional development of human B cells^25^. In athymic nude rats that lack T cells, B cells are similarly impaired but can be rescued by reconstituting adult rats with T cells^59^. It is not clear how T cells influence the development and maturation of other immune cells but interactions between T cells and progenitor cells in the bone marrow may be essential^60,61^.

Our data indicate that it takes ~4 months post-engraftment before T cell numbers stabilize in the blood of hNSG mice. This delay in human T cell development could explain why some studies that used hNSG mice too soon after engraftment have concluded that the human immune system of hNSG mice is not functional^13–16^. By 4 months post-engraftment, we found that innate and adaptive human immune cells were functionally competent; i.e., they responded *ex vivo* and *in vivo* to physiologically relevant stimuli by producing a range of immune effector molecules (e.g., cytokines, antibodies). These data are consistent with other data showing that inflammatory stimuli elicit robust effector functions in human immune cells from humanized mice (e.g., cytokine production, antibody synthesis, migration toward chemotactic cues, etc.)^2–11^. Overall, the composition of human immune cells in blood of hNSG mice is similar to that of standard mouse models, with most cells being T and B lymphocytes^62^. This differs from human blood, where most circulating immune cells are of myeloid origin. These species differences exict, in part, becuase mouse cytokines and growth factors cannot support the growth and development of human myeloid progenitor cells. “Next generation” humanized transgenic models do exist that replace mouse cytokines with their human equivalents and this enhances myeloid cell development but these mice die from progressive anemia and inappropriate immune activation^22,63,64^, severely limiting their usefulness in chronic studies of neuroinflammation and axon regeneration. Importantly, although human myeloid cells are sparse in the circulation of hNSG mice, they are abundant within the injured spinal cord of these mice if injured at 4-months post-engraftment, just as they are the injured spinal cord of wild-type mice and humans^32,40,44^.

We can infer from our comparative SCI studies that human immune system development and function are sub-optimal in hNSG mice at 2 months post-engraftment. Specifically, the magnitude of the neuroinflammatory response after SCI, as defined by numbers of intraspinal hCD45^+^ leukocytes and human T cells (hCD3, hCD4, hCD8), was markedly reduced in hNSG mice injured at 2 months post-engraftment relative to 4 months post-engraftment (Figs. 5 and 6). Notably, the magnitude of human T cell infiltrates and proportions of hCD4^+^ and hCD8^+^ subsets in the injured spinal cord of hNSG mice in the 4 month cohort were consistent with what is observed in conventional C57BL/6 and C57BL/10 mice after SCI^32,40^. The proportion of hCD4^+^ and hCD8^+^ subsets in the injured spinal cord reflected proportions in the blood, and SCI did not alter the proportion of human T cell subsets^65,66^. However, SCI preferentially decreased the number of cytotoxic T cells expressing CXCR3, a key chemokine receptor involved in T cell recruitment to sites of inflammation and autoimmunity^9,67,68^.

Many large, phagocytic human macrophages expressed membrane hCD4 within the lesion. Rat CD4^+^ macrophages are found at sites of myocarditis^69^, in tumors^38^ and in the injured rat spinal cord^32,33^. In the mouse gut, CD4^+^ macrophages also have unique ontogeny and function^70,71^. The CD4 co-receptor, which is routinely used as a phenotypic marker of helper T-cells, is expressed by human and rat monocytes^31,62^. Ligating this co-receptor on monocytes/macrophages triggers intracellular signaling cascades that augment macrophage maturation and activation^35,72^. Although the functional significance of CD4^+^ macrophages in CNS trauma has not been determined, macrophage expression of CD8, another co-receptor typically found on cytotoxic T cells, has been linked to pathological effector functions and pathology in the rat models of multiple sclerosis^34^, stroke^39^, and spinal cord injury^33^. Therefore, humanized mice could prove to be a useful tool for studying the functional importance of activating CD4 and other human-specific co-receptors on CNS macrophages.

We found several examples of cell-cell contact between human T cells and human macrophages in the injured spinal cord. Membrane hCD3 expression was abundant at the interface between human macrophages and human T cells (see Supplemental Fig. 7); redistribution of CD3, a component of the T cell receptor (TCR) complex, is essential in the formation of immune synapses and T cell activation by macrophages and antigen presenting cells^73^. Importantly, human macrophages within the injured spinal cord of hNSG mice expressed human MHC class II antigen complexes (HLA-DR/DP/DQ). Functionally, MHC class II is required for effective antigen presentation and activation of T cells, and its expression on macrophages increases in response to select chemokines and cytokines. Although we did not directly measure intraspinal T cell activation in injured hNSG mouse spinal cord, data in this report indicate that the presence of mature functional human T cells and macrophages is linked to worsened lesion pathology and impaired functional recovery. We cannot discount the possibility that some human immune cells may exert neurotrophic or protective effects in the lesion. Indeed, axons sprout in close proximity to human immune cell foci in hNSG mice^1^. This could be a result of the developmental age of the human immune system; however, adult mouse and rat macrophages and T cells have been shown to have both injurious and neuroprotective effects after SCI^43,47,50,54,57,74–82^. To prove a causal role for human T cell-mediated pathology or repair, further studies are needed in which human immune cells are manipulated (e.g., activated or depleted).

It is still unclear whether aging affects the health and viability of humanized mice. We consistently kept hNSG mice alive for 9–12 months without developing major health problems. Sporadic premature death of hNSG mice does occur but cause of death is difficult to determine. Anemia may be one factor contributing to premature mortality^22,63,64^. New transgenic mouse models with improved engraftment and maturation of human innate immune cells typically die 3–4 months after engraftment because of rapid onset anemia^22^. Other reports indicate that progressive destruction of mouse RBCs can occur with high levels of human engraftment^22,63,64^. SCI in hNSG did not increase mortality rates, although we did not allow survival beyond 35 days post-SCI. While hNSG mice can develop infrequent health problems (e.g., ruffled fur, cataracts in one or both eyes and spontaneous hindlimb inflammation), SCI did not increase the number or severity of these health issues.

A criticism of humanized mice is that they do not produce robust human immune responses *in vivo*^13–16^. As detailed above, the belief that humanized mice are immunologically impaired has been perceived as a weakness of the model but in reality, when assays are performed at later post-engraftment intervals, human immune cells respond as expected^6,25,26^. In our hands, a stable human immune system requires at least 3–4 months to develop in hNSG mice. This process may be affected by factors that vary between labs, including strain of the recipient immune deficient mice, age of engraftment (newborn vs. young adult mice), route of human stem cell injection (intraperitoneal vs. intravenous vs. intrahepatic), human stem cell source (UCB vs. fetal liver vs. adult bone marrow), stem cell phenotype (cell surface expression of CD34, CD38 and CD133), stem cell purity, stem cell dose, supplements given during development (e.g., rhIL7), engraftment of additional human tissues (fetal liver/thymus), etc. Each of these variables should be considered when interpreting published data; however, none are disqualifying factors that would preclude the use of humanized mice in experimental models of CNS trauma or neuroinflammation.

Although there are several humanized mouse models, many that include “next generation” strategies for manipulating the composition and function of the human immune system in mice (see Shultz *et al*.^83–85^ for review), we used the hNSG model because it is the current standard for long-term studies across a range of scientific disciplines^3,6,12,24–26,86^. Moreover, hNSG mice are available through commercial vendors, although current costs may be prohibitive for larger studies (~$1,000 USD/mouse). Fortunately, it is possible to reduce costs and generate hNSG mice “in house”. In our experience, it is possible to generate a litter of 8–10 hNSG mice for ~$1,500 USD – this cost includes the purchase and housing of breeding pairs, a single vial of human UCB stem cells (each vial contains ~5×10^5^ cells) and housing per-diem costs for ~6 months. The final per mouse cost is ~$150–200 USD. Generating your own hNSG mice also make it possible for sex to be incorporated as a biological variable in the design of experiments^87^; commercial vendors only provide ready to use cohorts of female mice.

Data in the current report indicate that a consistently functional human immune system will develop in immune compromised mice ~4 months after engraftment. Once activated by SCI, human immune cells infiltrate the injured spinal cord where, through unknown mechanisms, they exacerbate lesion pathology and impair recovery of function. Using data and guidelines established in this report, future research can incorporate humanized mice into experimental plans to better understand how SCI, or other types of neurological injury and disease, affect human immune system function *in vivo*. In this context, humanized mice provide a unique tool in the translational research toolbox and should facilitate a better understanding of human immune cell-mediated injury and repair mechanisms and the *in vivo* testing of human-specific immunomodulatory interventions before advancing individual therapies into human clinical trials.

## Methods

### Humanized mice

The Institutional Animal Care and Use Committee of the Office of Responsible Research Practices at The Ohio State University approved all animal protocols. All experiments were performed in accordance with the guidelines and regulations of The Ohio State University and outlined in the Guide for the Care and Use of Laboratory Animals from the National Institutes of Health. Adult male and female NOD.Cg-Prkdc^scid^IL2rg^tm1Wjl^/SzJ (NSG mice) breeding pairs were purchased from The Jackson Laboratory (strain #005557) and bred in-house. Animals were fed commercial food pellets and chlorinated reverse osmosis water ad libitum. Mice were housed ≤5/cage in ventilated microisolator cages containing with corn cob bedding, and the housing facility in a 12-hour light-dark cycle at a constant temperature (20 ± 2 °C) and humidity (50 ± 20%). NSG strain mice were kept in sterile housing conditions during breeding and throughout the humanization process. Generation of NSG mice with human immune systems (hNSG) was performed as previously described^1,28,88^. NSG pups received 100 cGy whole body irradiation (RS 2000, Rad Source, Suwanee, GA) 24–72 hours postnatal, followed immediately by engrafting 1–5 × 10^4^ human umbilical cord CD34^+^ stem cells (Lonza Incorporated, Walkersville, MD; or Stemcell Technologies, Vancouver, BC) via intrahepatic injection. After injection pups were allowed to recover for several minutes on a heat source set to 37 °C before returning pups to their dams for normal maturation and weaning at 21–24 days of age. hNSG mice are healthy and can be used over extended periods of time for experimentation. On occasion, hNSG mice can have a rough coat and decreased body weight compared to non-humanized NSG mice. Cataract formation in one or both eyes was also observed in a few animals, and 1/78 hNSG mice developed spontaneous hindlimb inflammation around the knee. Median survival of our initial cohort of hNSG mice was 293 days. While death was preceded by rapid weight loss over ~1 week, there were no phenotypic changes associated with classical graft-versus-host disease (GvHD), such as loss of fur and advanced infiltration of cells into organs and tissues. Only 4% (3/78) of hNSG mice displayed evidence of T-cell expansion (excessive thymus size or proportion of T cells in peripheral blood), further supporting a lack of graft-versus-host disease (GvHD) in this model. Both male and female hNSG mice generated robust human immune systems. The efficiency and composition of engrafted human immune cells differed only slightly between male and female NSG mice in blood at 4 months post-engraftment. Total engraftment by human immune cells was 7.5% higher in females (63.0 ± 14.2% vs. 55.5 ± 17.2%), with more B cells (67.2 ± 16.3% vs. 56.2 ± 17.6%) and fewer myeloid cells (3.91 ± 2.8% vs. 6.73 ± 3.8%). Overall, these data support the use of both male and female humanized mice in experimental studies.

### SCI & animal care

hNSG mice (2–4 months old) were used for SCI experiments. hNSG mice used for comparison of time post-engraftment were generated 2 months apart using same UCB donor but underwent SCI and all other outcomes at the same time. Mice were anesthetized with ketamine (90–120 mg/kg, i.p.) and xylazine (7–10 mg/kg, i.p.) then injected with gentamicin sulfate (5 mg/kg, s.q.). Aseptic conditions were maintained during all surgical procedures. Mice were placed on a warming pad during surgery to maintain body temperature. Hair was shaved at the region of the thoracic spinal cord and skin treated with a sequence of betadine-70% ethanol-betadine. A small midline incision was made to expose the mid-thoracic vertebra, then a partial laminectomy was performed. A 60 KDyne (mild) T9 contusion injury was performed using an Infinite Horizon Impactor (Precision Systems and Instrumentation, Lexington, KY). Immediately after impact, the muscle overlaying the injury site was sutured, followed by closure of the wound with sutures or staples. After surgery, mice were placed in cages on heating pads and monitored until they recovered consciousness. Animals were supplemented with sterile saline (1–2 mL, s.q.) and softened food to eat *ad libitum* during recovery. Bladders were expressed twice daily, and urine underwent weekly pH testing to detect bladder infections. Gentocin antibiotic was subcutaneously administered once a day at 5 mg/kg for 5 days post-injury (dpi).

### Hindlimb locomotor function assessment

The open-field Basso Mouse Scale (BMS) was used to assess hindlimb paralysis and functional recovery^89^. Pre-SCI testing occurred after acclimating mice to the open field on multiple days with dim lighting to minimize anxiety. Post-SCI testing occurred on 1, 3, 5, 7, 14, 21, 28, and 35 dpi. Mice explored the open field and were scored over a period of 4 minutes. Two individuals that were blinded to the experimental condition averaged the left and right hindlimb score for each mouse to obtain a single score per mouse at each time point.

The horizontal ladder test was performed before SCI and at 35 dpi. The horizontal ladder contained 33 rungs spaced ~1 cm apart and elevated ~1 cm off of a clear mirrored surface for viewing of paws from the side and below using a digital video recorder to capture each trial. Mice were acclimated to the ladder test over 3 separate days with a minimum of 3 runs per session before acquiring baseline values. Mice were handled by a single experimenter (RSC). During testing days, each mouse was allowed a minimum of 3 passes along the horizontal ladder to reduce trial-trial variability. Mice were allowed to rest for ~15–20 minutes between each pass/trial. Digital videos were assessed by a single experimenter (RRJ) blinded to group designations, with playback at 0.35× speed using VLC media player (v.3.0; VideoLAN Organization) on a computer screen of a minimum 13-inch diameter. Each trial (3/mouse) was viewed and analyzed 3 times by the experimenter and averaged to acquire a single value for each trial. This was done to reduce observer error and variability, and a subset of trials were assessed by a second reviewer (RSC) to ensure accuracy.

### Tissue collection

For survival blood sampling, blood was collected from the submandibular vein using a lancet and EDTA-coated capillary tube (Sarstedt Inc., Thermo Fisher Scientific, Waltham, MA). For endpoint tissue collection, mice were first deeply anesthetized with ketamine and xylazine. Blood was collected via cardiac puncture, placed in EDTA-coated collection tubes, and inverted several times. Red blood cells were lysed with ammonium chloride and resuspended in 0.1 M phosphate buffered saline (PBS) with 2% FBS (flow buffer) for flow cytometry. Spleens were isolated, weighed, and placed in Hank’s Balanced Salt Solution (HBSS). Spleens were minced with sterile dissection scissors and smashed through a 40 μm sterile filter using the plunger of a 3 mL syringe and 10 mL of HBSS. Mouse femurs and tibiae were removed, cleaned, and placed in a small volume of HBSS. Bone marrow cells were isolated by either flushing bones with 10 mL HBSS or by crushing in a mortar and pestle with HBSS. Cell counts for bone marrow and spleen were obtained by standard hemocytometer counting techniques, while total blood was analyzed with a Hemavet 950 fs multi-species hematology system capable of analyzing whole blood cells with 5-part differential, platelets, and red blood cells. To obtain serum, blood was collected as above, inserted into gel separation tubes coated with clotting activator, and centrifuged at 10^5^ × g for 5 minutes. For experiments using tissue sections, mice were perfused with 0.1 M PBS followed by 4% paraformaldehyde. Fixed tissues (i.e., the spleen, thymus and spinal cords) were cryopreserved for 48 hours in 30% sucrose, cryosectioned at 10 μm onto glass slides, and stored at −20 °C until staining.

### Immunolabeling and imaging

Antibody sources, working concentrations, protocols, and validations were previously reported^1^; a summary of antibodies for immunolabeling are included in Table 1. Slides were thawed on a slide warmer and then rinsed in 0.1 M PBS. Endogenous peroxidases were quenched with 6% H_2_O_2_ diluted in methanol for 15 minutes at room temperature. Sections were incubated with 4% BSA, 3% normal goat serum, and 0.1% Triton X-100 in 0.1 M PBS for 1 hour followed by primary antibody overnight at 4 °C. Secondary antibody incubation was performed at room temperature for 1 hour. Slides were then incubated with Vectastain ABC (Vector Laboratories, Burlingame, CA) for 1 hour at room temperature and developed with 3,3′-diaminobenzidine (DAB/ImmPACT DAB) or Vector SG (Vector Laboratories, Burlingame, CA) of for 5–15 minutes (until optimal differentiation of signal). A separate protocol was used to reduce non-specific labeling of mouse antibodies on mouse tissue to identify human immune cell types. After thawing, tissue sections were placed in ice-cold methanol for 15–20 minutes, followed by washes in PBS. When required (i.e. hCD3, hCD8, hCD4), antigen retrieval was accomplished by incubating tissues in heated alkaline pH Tris-based solution (Vector Laboratories) followed by a blocking step using a mixture of 4% BSA, 3% normal goat serum and 0.1% Triton X-100 in 0.1 M PBS for 1 hour. Sections were incubated with primary antibodies overnight at 4 °C, followed by washes in PBS and subsequent incubation at room temperature for 1 hour with Alexa Fluor-conjugated secondary antibodies. Immunolabeled tissues were imaged using an Axioplan 2 imaging microscope equipped with an AxioCam digital camera and AxioVision v.4.8.2 software (Carl Zeiss Microscopy GmbH, Jena, Germany), or on a Leica TCS SCP confocal microscope (Leica Microsystems Inc., Buffalo Grove, IL).

**Table 1:** Antibodies.

Antigen	Host, dilution	RRID	Vendor, catalog number
Primary antibodies			
GFAP	Rabbit, 1:5000	AB_10013382	Agilent, Z0334
hCD45	Mouse, 1:250	AB_400305	BD Biosciences, 347460
hCD3	Rat, 1:250	AB_321245	Bio-Rad, MCA1477
hCD4	Rabbit, 1:500	AB_2686917	Abcam, ab183685
hCD8	Mouse, 1:250	AB_395995	BD Biosciences, 555631
HLA-DR/DP/DQ	Mouse, 1:100	AB_794857	Thermo Fisher Scientific, MA1-25914
Secondary amplification/detection			
Biotin-rabbit IgG	Goat, 1:1000	AB_2313606	Vector Labs, BA-1000
Biotin-rat IgG	Rabbit, 1:1000	AB_10015300	Vector Labs, BA-4001
Alexa488-rabbit IgG	Goat, 1:500	AB_2576217	Invitrogen, A-11034
Alexa546-mouse IgG	Goat, 1:500	AB_144695	Invitrogen, A-11030
Alexa633-rat IgG	Goat, 1:500	AB_141553	Invitrogen, A-21094
Flow cytometry antibodies (per test)			
mFc Block, CD16/32	Rat, 1 μL	AB_394657	BD Biosciences, 553142
hFc Block	Unknown, 1 μL	AB_2728082	BD Biosciences, 564219
mCD45-APC	Rat, 2.5 μL	AB_398672	BD Biosciences, 559864
hCD45-PerCP	Mouse, 10 μL	AB_400307	BD Biosciences, 347464
hCD3-APC-H7	Mouse, 4 μL	AB_1645731	BD Biosciences, 641397
hCD3-Pe-Cy7	Mouse, 5 μL	AB_396896	BD Biosciences, 557851
hCD4-APC	Mouse, 4 μL	AB_314082	BioLegend, 300514
hCD4-PerCP-Cy5.5	Mouse, 3 μL	AB_400451	BD Biosciences, 341653
hCD8-Pe-Cy7	Mouse, 4 μL	AB_314130	BioLegend, 301012
hCD19-BV510	Mouse, 5 μL	AB_2737912	BD Biosciences, 562947
hCD33-FITC	Mouse, 10 μL	AB_400047	BD Biosciences, 340533
hCD69-BV421	Mouse, 3 μL	AB_10933255	BioLegend, 310929
hCXCR3-BV421	Mouse, 3 μL	AB_2737653	BD Biosciences, 562558
hIFNγ-FITC	Mouse, 3 μL	AB_394516	BD Biosciences, 552887

### Lesion pathology

Glial Fibrillary Acidic Protein (GFAP) labeled sections were imaged using a 5× objective. Images were loaded into ImageJ, and a random dot overlay (each dot representing 0.01 mm^2^) was placed on the image using the grid feature. Total number of dots per section, and number of dots within the GFAP-negative region, was determined and the proportion of the section area and total lesion volume calculated. Human CD3^+^ T cells within the lesions of hNSG mice were manually quantified on an Axioplan 2 microscope with a 20× objective. Two sections at the lesion epicenter (100 μm apart) were quantified, and values were averaged for each animal. Images of T cell subsets (hCD3^+^/hCD4^+^ and hCD3^+^/hCD8^+^) were acquired with a Leica TCS SCP confocal microscope with 20× magnification. Tiling with automatic stitching generated a high-resolution image covering the whole section, and imaging parameters were kept consistent to minimize variability. Z stacks (2 μm steps) were compressed to create one maximum intensity projection (MIP) image. Because T cells predominantly home to the gray matter^40^, and the spared white matter contained high background staining, only spinal lesion and gray matter was included in the analysis. First, a mask of the lesioned gray matter was created using the freehand selection tool in ImageJ. Masks of the lesioned gray matter were imported into MIPAR software^90^. T cells were counted using custom cell counting algorithms (available upon request). Briefly, for quantification of human T helper cells (hCD3^+^/hCD4^+^), each channel was selected to generate two layers and a basic threshold for staining intensity was applied to each layer. Cells >50 pixels in size were removed from each layer and touching cells were split. Cells meeting stain and size criteria with spatially overlapping channels counted as one helper T cell. Batch processing applied this algorithm to all images, and the cell count data was exported to Microsoft Excel. This method was repeated for counting of human cytotoxic (hCD3^+^/hCD8^+^) T cells.

### Flow cytometry

1 × 10^6^ bone marrow cells and splenocytes, or approximately 50 μL RBC-lysed blood, were allocated for flow cytometry analysis. Human and mouse Fc receptors were blocked for 15 minutes, followed by labeling with antibodies against specific antigens for 1 hour. A summary of antibodies and amounts added to samples for flow cytometry are included in Table 1. Dead cells were labeled with eFluor780 (eBioscience, Thermo Fisher Scientific) approximately 30 minutes into antibody incubation. Labeled cells were fixed and permeabilized with BD Fix/Perm solution (BD Biosciences, Franklin Lakes, NJ) for 20 min. To identify IFNγ^+^ human T cells, cells were immediately fixed and permeabilized after stimulation, followed by staining in BD Perm/Wash buffer using Human Th1/Th2/Th17 Phenotyping kit (BD Biosciences cat #560751). All incubations were performed at 4 °C and followed by a wash with excess flow buffer and centrifugation for 5 minutes at 4 °C. An LSR II flow cytometer (BD Biosciences) analyzed immunolabeled cell samples. Forward scatter and side scatter parameters gated viable cell populations for phenotypic analysis using antibody panels. Offline data analyses were completed with FlowJo v.10 software (Tree Star, Inc., Ashland, OR).

### *In vivo* lipopolysaccharide challenge

hNSG and non-engrafted NSG mice (4–6 months old) were injected 3 mg/kg (i.p.) with lipopolysaccharide (LPS from *E*. *coli*; O55:B5, Sigma-Aldrich) or 0.9% saline solution. Mice were anesthetized, blood collected for serum, and tissues placed in 4% PFA overnight at 4 °C. Blood was collected into a BD Microtainer serum separator tube with clotting activator, and after 30 minutes tubes were centrifuged at 10,000×g for 5 minutes. Serum was collected, aliquoted into 1.5 mL Eppendorf tubes, and stored at −80 °C.

### *Ex vivo* purification and stimulation of human splenocytes

hNSG mice (4–6 months old) were anesthetized and spleens dissected as previously described. Splenocytes were diluted in IMDM and counted on a manual hemocytometer with a 1:1 ratio of 0.4% Trypan Blue to quantify live cells. Contaminating mouse splenocytes were depleted from cell preparations by incubating with MACS anti-mouse CD45 magnetic microbeads (Miltenyi Biotec, Auburn, CA) and then washing through magnetic LD Columns as per manufacturer’s protocol. Enrichment of human cells was verified via flow cytometry of the depleted and cultured fractions: depleted fraction contained >90% mouse CD45^+^ splenocytes, while the cultured fraction contained >90% human CD45^+^ splenocytes (see Supplemental Fig. 4).

Prior to culture and stimulation, human splenocytes were stained with CFSE using CellTrace CFSE Cell Proliferation Kit (Invitrogen) as per manufacturer recommendations. Human splenocytes were then cultured at 1×10^6^ cells per mL in ImmunoCult-XF T cell Expansion Medium (Stemcell Technologies, Vancouver, BC). For T cell stimulation, ImmunoCult Human CD3/CD28 T cell Activator and 20 ng rhIL2 (Stemcell Technologies) was added to culture medium. For B cell stimulation, anti-human CD40 antibody (clone 5C3) and 20 ng rhIL4 (Stemcell Technologies) was added to culture medium. For LPS stimulation, 1 μg/mL was added to culture medium. Splenocyte cultures were maintained at 37 °C in a 95% air and 5% CO_2_ incubator for 48–96 hours.

Spleens were isolated from hNSG mice 35 days after SCI for quantification of IFNγ^+^ human T cells. Splenocytes were collected, resuspended at 1×10^6^ cells per mL in RPMI 1640, and stimulated with Cell Activation Cocktail (PMA/Ionomycin cat #423301; BioLegend, San Diego, CA) in the presence of BD GolgiStop Protein Transport Inhibitor containing monensin for 6 hours at 37 °C in a 95% air and 5% CO_2_ incubator. Activated cells were then harvested and stained as previously described.

### Quantification of cytokines and antibodies from blood serum and culture supernatants

Human TNFα was quantified in blood serum (1:10 dilution) using a DuoSet ELISA kit (R&D Systems, Minneapolis, MN). Human IFNγ and IL10 were quantified in culture supernatants (1:2 dilution) using DuoSet ELISA kits (R&D Systems). DuoSet ELISA Ancillary Reagent Kit 2 was used in conjunction with all DuoSet ELISA kits. Human IgG and IgM was quantified in blood serum (1:1000 dilution) and culture supernatants (1:10 dilution) using the Human IgG/IgM total ELISA Ready-SET-Go! Kits (Invitrogen/eBioscience). All ELISAs were performed as per manufacturer’s protocols. Plates were read at 450 nm wavelength on a SpectraMAX190 and analyzed using the SoftMax Pro software (Molecular Devices, San Jose, CA).

### Statistical analysis

GraphPad Prism software (version 5.0, La Jolla, CA) was used for data analysis. Statistical significance was assigned to a p < 0.05. Statistical tests are reported in figure legends and include unpaired t-test (with or without Welch’s correction for unequal variance), one-way ANOVA (with Tukey’s post-hoc test), repeated-measures two-way ANOVA (with Bonferroni post-hoc test), and Kaplan-Meier survival curve estimation. Data are represented as mean ± standard error of the mean except where noted. One sample from both 2- and 4-month post-engraftment groups was not run on the Hemavet due to inadequate blood volume at 35 dpi and was excluded from analysis in Fig. 4C,D. Illustrations were created with BioRender (BioRender.com). Figures were generated with Adobe Photoshop CS5 v.12 (Adobe Systems Inc., San Jose, CA).

## Supplementary information

Supplementary information

## Data Availability

All data from the current study are available by reasonable request from the corresponding author.
