# Supplementary material for: Human immune cells infiltrate the spinal cord and impair recovery after spinal cord injury in humanized mice
Source: Sci Rep. 2019 Dec 13;9:19105. doi: 10.1038/s41598-019-55729-z (PMC6911055; doi:10.1038/s41598-019-55729-z)
Supplement: Supplementary file 1 — Supplementary information [file 41598_2019_55729_MOESM1_ESM.pdf]

Supplemental Figures

Human immune cells infiltrate the spinal cord and impair recovery after spinal cord injury in humanized mice

Randall S. Carpenter[1-4], Roselyn R. Jiang[2-4], Faith H. Brennan[2-4], Jodie C.E. Hall[2-4], Manoj K. Gottipati[2-4], Stefan Niewiesk[5] and Phillip G. Popovich[2-4]

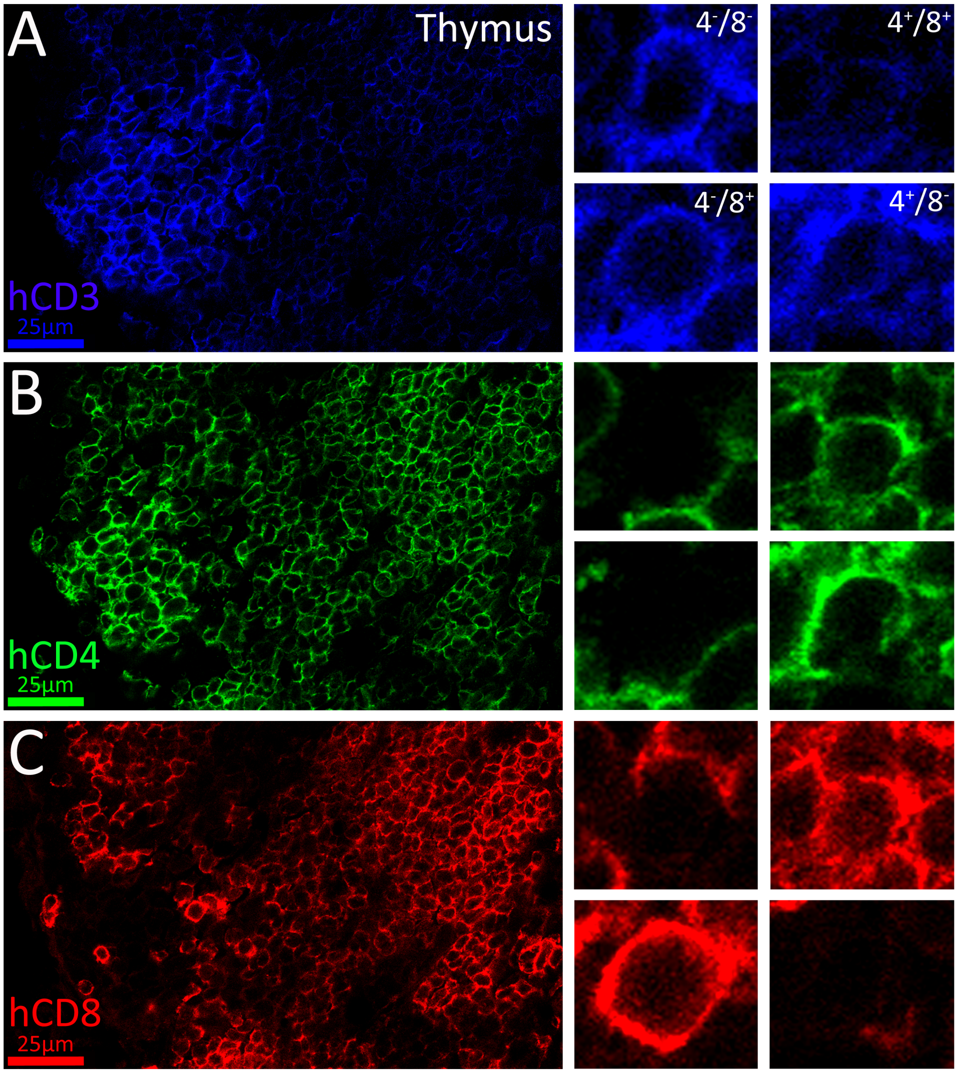

Supplemental Figure 1. Isolated fluorescent channels from confocal images used in Figure 1F demonstrating human CD3 (A), human CD4 (B), and human CD8 (C) immunolabeling in the thymus of a naïve hNSG mouse 4-months post-engraftment. Human T cell subsets in thymus are highlighted with high-resolution confocal microscopy.

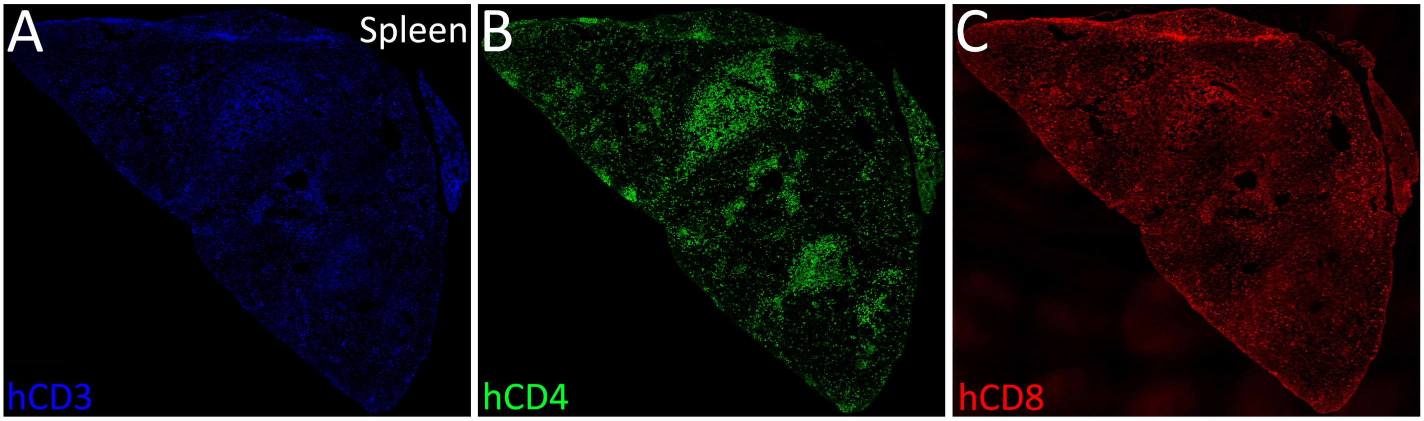

Supplemental Figure 2. Isolated fluorescent channels from low-magnification confocal image used in Figure 1G demonstrating human CD3 (A), human CD4 (B), and human CD8 (C) immunolabeling in the spleen of a naïve hNSG mouse 4-months post-engraftment.

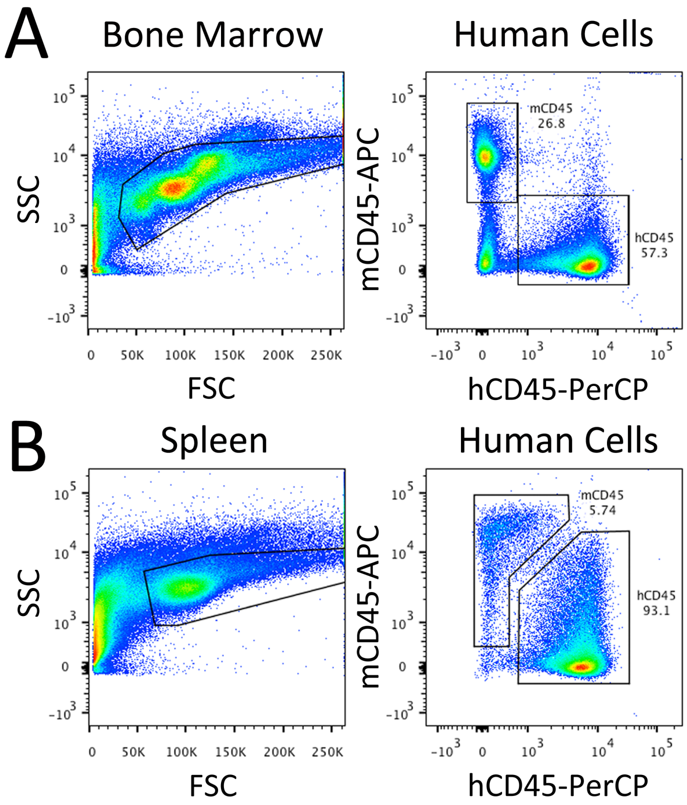

Supplemental Figure 3. Example flow cytometry plots identifying human CD45$^+$ immune cells in bone marrow (C) and spleen (D) of a naïve hNSG mouse 4 months post-engraftment. Left plots demonstrate gating of cells by size (FSC = forward scatter) and complexity (SSC = side scatter. Right plots demonstrate gating of mouse and human CD45$^+$ immune cells using fluorescently labeled antibodies.

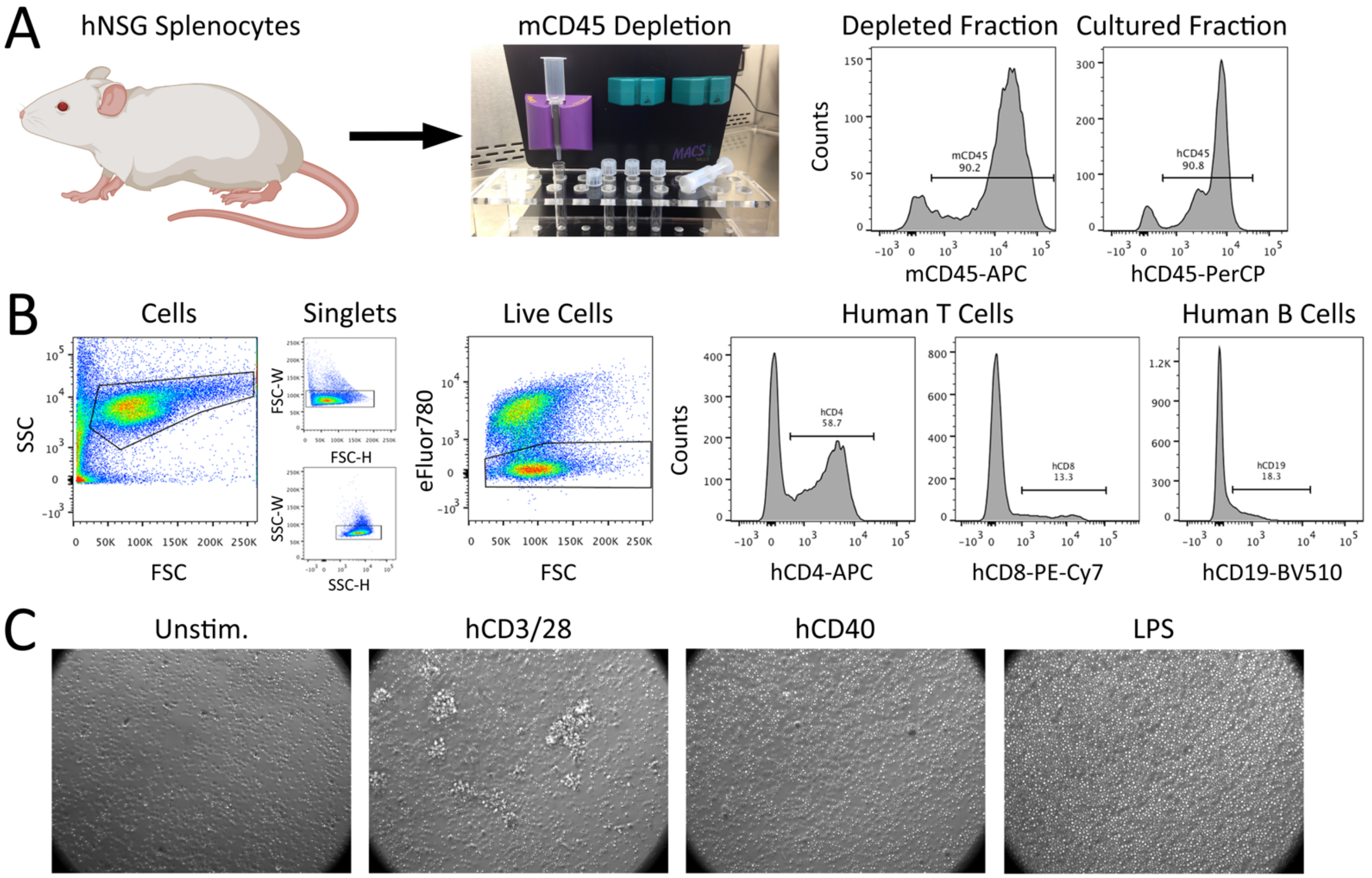

Supplemental Figure 4. Isolation and flow cytometric analysis of human splenocytes from hNSG mice. A) Mouse splenocytes were depleted from hNSG splenocyte preparations using Miltenyi MACS magnetic bead depletion of mouse $CD45^+$ cells. Flow cytometry confirmed mouse splenocyte depletion, with >90% of depleted cells expressing mouse CD45, and >90% of cultured cells expressing human CD45. Human splenocytes were then left unstimulated (unstim) or stimulated with either lipopolysaccharide (LPS), human CD40 monoclonal antibody with rhIL4, or human CD3/28 antibody with rhIL2. B) Example flow cytometry analysis of single cells after 48-96 hours in culture, including $hCD4^+$ and $hCD8^+$ T cells, and $hCD19^+$ B cells. C) Low-magnification images of culture conditions after 48 hours. Note the appearance of proliferating cell clusters in the hCD3/28 culture well.

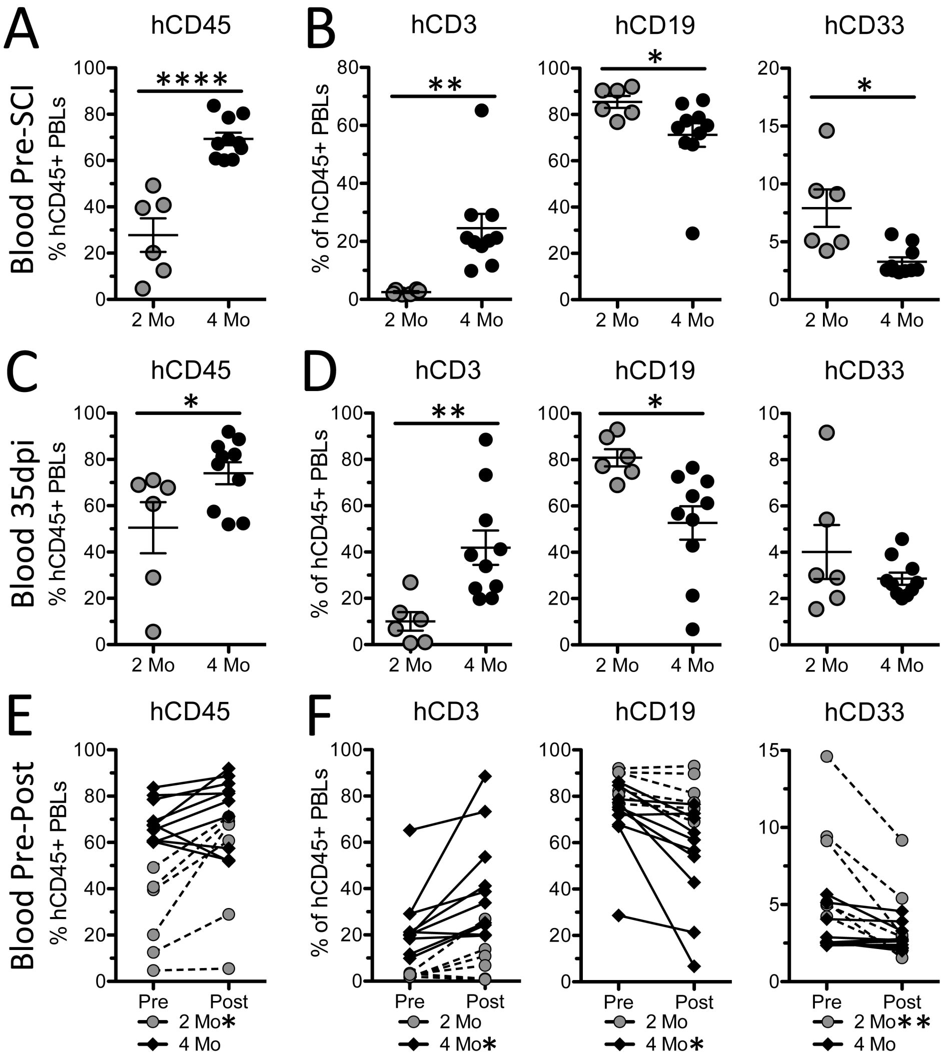

Supplemental Figure 5. Human peripheral blood leukocyte responses to SCI differ as a function of time post-engraftment. Proportion of human PBLs (A,C), and human PBL subsets (B,D), 7 days prior to and 35 days after SCI. Change in proportion of human PBLs (E), and human PBL subsets, from pre- to post-SCI. *p<0.05 **p<0.01 ****p<0.0001 student's unpaired (A-D) and paired (E,F) t-test. Data average ± SEM.

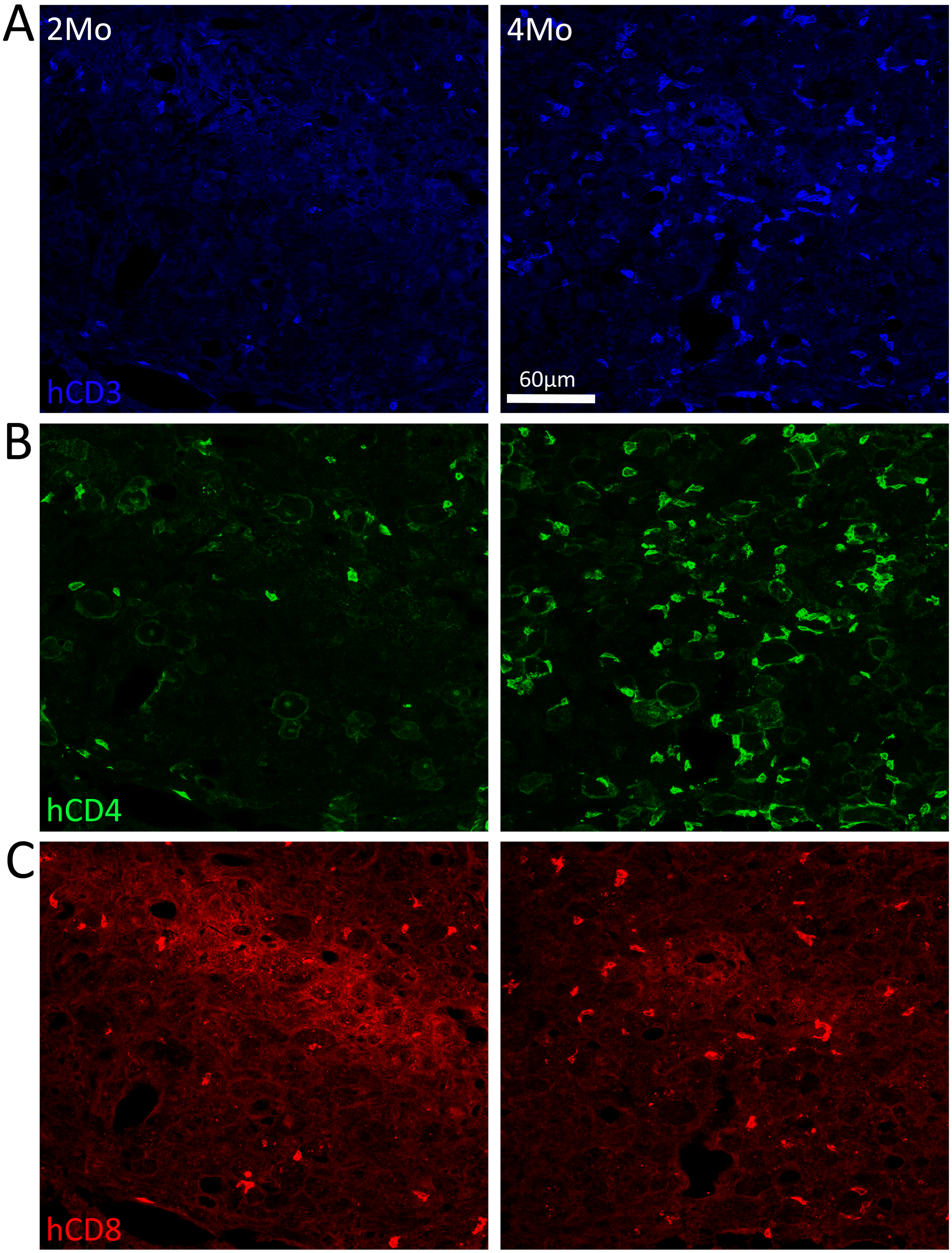

Supplemental Figure 6. Isolated fluorescent channels from confocal images used in Figure 6A demonstrating human CD3 (A), human CD4 (B), and human CD8 (C) immunolabeling in lesion epicenters 35 dpi in hNSG mice injured at either 2- or 4-months post-engraftment.

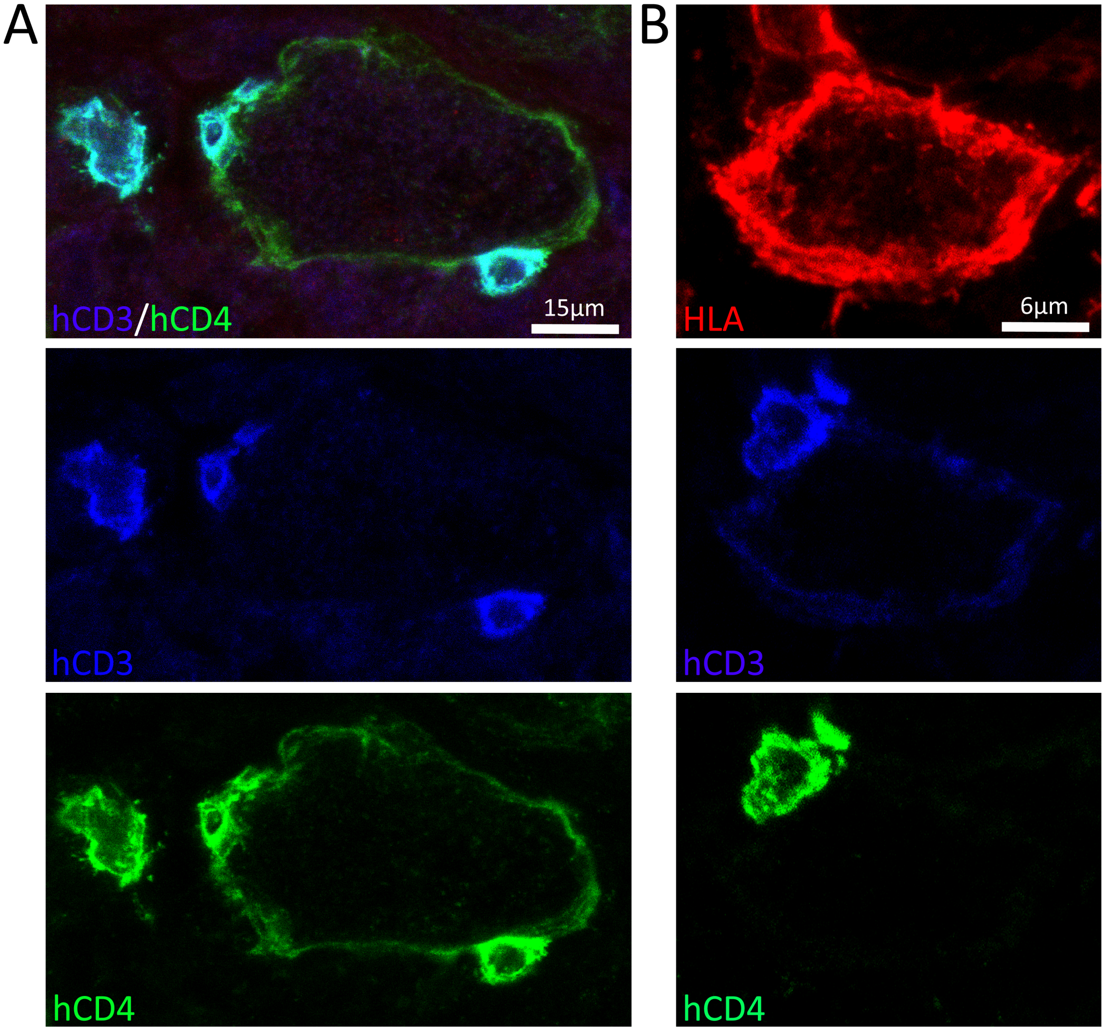

Supplemental Figure 7. A) Combined and Isolated fluorescent channels from confocal image used in Figure 7B demonstrating human CD3 and human CD4 immunolabeling in the lesion epicenter 35 dpi in a hNSG mouse injured at 4-months post-engraftment. The co-localization of hCD3 and hCD4 on small cells confirms their T cell identity. In this example, two human T cells are in direct contact with a human CD4$^+$ macrophage. Note the absence of hCD3 labeling on the human macrophage. A large, isolated T cell with immunoblast-like morphology is also identified. B) Isolated fluorescent channels from confocal image used in Figure 7C demonstrating a human helper T cell contacting an HLA$^+$ human macrophage. The CR3/43 antibody clone identifies human HLA-DR/DP/DQ complexes involved in antigen presentation.
